# Synthesis, Crystal Structure, Spectroscopic Properties, and Interaction with Ct-DNA of Zn(II) with 2-Aminoethanethiol Hydrochloride Ligand

**DOI:** 10.1155/2016/2691253

**Published:** 2016-02-10

**Authors:** Xu-gang Shu, Chun-li Wu, Cui-jin Li, Min Zhang, Ke Wan, Xin Wu

**Affiliations:** ^1^College of Chemistry and Chemical Engineering, Zhongkai University of Agriculture and Engineering, Guangzhou 510225, China; ^2^College of Light Industry and Chemical Engineering, Guangdong University of Technology, Guangzhou 510006, China; ^3^Key Laboratory of Agro-Ecological Processes in Subtropical Region, Institute of Subtropical Agriculture, Chinese Academy of Sciences, Changsha 410125, China

## Abstract

The zinc(II) complex (C_2_H_6_NS)_2_Zn·ZnCl_2_ was synthesized with 2-aminoethanethiol hydrochloride and zinc sulfate heptahydrate as the raw materials in aqueous solution. The composition and structure of the complex were characterized by elemental analysis, infrared spectra, single crystal X-ray diffraction, and thermogravimetry. The crystal structure of the zinc(II) complex belongs to monoclinic system, space group *P*  2_1_/*n*, with cell parameters of *a* = 0.84294(4), *b* = 0.83920(4), *c* = 1.65787(8) nm, *Z* = 2, and *D* = 2.041 g/cm^3^. In this paper, the interaction of complex with Ct-DNA was investigated by UV-visible and viscosimetric techniques. Upon addition of the complex, important changes were observed in the characteristic UV-Vis bands (hyperchromism) of calf thymus DNA and some changes in specific viscosity. The experimental results showed that the complex is bound to DNA intercalative (intercalation binding).

## 1. Introduction

In recent years, the application of 2-aminoethanethiol hydrochloride, a cysteamine alternative, has garnered widespread attention [1–3]. However, cysteamine itself is not stable and easily undergoes metamorphosis due to the direct absorption of other substances. The researcher considered the fact that 2-aminoethanethiol hydrochloride structure contains NH_2_ and SH, which synthesize the complex of cysteamine hydrochloride with zinc(II) ions, and the complex ratio of the cysteamine hydrochloride to zinc is approximately 2 : 1 [4]. The zinc(II)-organic framework of cysteamine combined with zinc(II) ions is similar to a zinc(II) amino acid chelate, which has dual nutritional and therapeutic effects [5–7]. Among various microelement amino acid chelates, the complex of cysteamine with zinc(II) ions will receive more attention because of the ligand's peculiarity in biology and widespread use in various medical treatments [8–13]. In the field of bioinorganic chemistry, the interaction between inorganic metal complexes and DNA has been an important area of research. In the last few years, the investigation of complex-DNA interaction is important to understand the biological activity of the complex in molecular level, indicating the interaction specificity of complex with DNA [14–16]. Because the ligand of cysteamine is involved in energy transduction and it controls metabolic processes and affects the mRNA expression of BCL-2, BCL-XL, MCL-1, BAX, and BID in organisms by participating in various enzymatic reactions [12, 13], studying the interaction of complex of cysteamine combined with zinc(II) ions with DNA has vital significance.

Based on zinc sulfate heptahydrate and 2-aminoethanethiol hydrochloride as the main synthetic raw materials, zinc(II) complex was obtained by altering the reaction conditions. We report herein the X-ray single crystal structure of zinc(II) complex with cysteamine that was synthesized in chemical reactions at appropriate temperatures, and the complex ratio of the cysteamine hydrochloride to zinc is 1 : 1. Moreover, the complex was characterized by elemental analysis, Fourier transform infrared spectroscopy (FT-IR), and thermogravimetric analysis (TG), as a continuation of our research directed to study the interaction of complex and calf thymus DNA (Ct-DNA) by UV-Vis absorption and viscosity measurements to understand the molecular mechanisms of them.

## 2. Experimental

### 2.1. Materials and General Methods

All of the chemicals were of analytical reagent grade. Solutions were prepared with distilled water; highly polymerized calf thymus DNA was purchased from Sigma. The stock solution of DNA was dissolved in 0.1 M of the Tris-HCl buffer (pH 7.2) and stored at 4°C. Zinc(II) was determined by EDTA complexometric titration. The content of cysteamine in the complex was measured by iodine stoichiometry titration. FT-IR and thermogravimetric analysis were determined in the Instrumental Analysis & Research Center of Sun Yat-Sen University. The IR spectra were obtained with a Perkin-Elmer Spectrum One spectrometer in the range of 400–4000 cm^−1^ using KBr pellets. Thermogravimetric analysis of the metal complexes was performed on a simultaneous STA 409 PC thermal analyzer, and the measurement was recorded from 30 to 800°C at a heating rate of 10°C min^−1^ under air flow of 100 mL min^−1^. Absorbance spectra were recorded using a spectrophotometer (Lambda 950). The absorbance measurements were performed by keeping the complex concentration constant (1.744 × 10^−4^ mol/L) while varying the Ct-DNA concentration (from 0 to 6 × 10^−5^ mol/L). The samples were incubated at 37°C for 15 min, and the spectra were recorded in the range of 200–600 nm. For viscosity measurements [17], a viscosimeter (SNB-2) measured with a digital stopwatch to evaluate the viscosity *η*′ of the samples. The data were reported as (*η*′/*η*′°)^1/3^ and [Complex]/[DNA], where *η*′° is the viscosity of the DNA solution alone.

### 2.2. Synthesis of the Compound

(C_2_H_6_NS)_2_Zn·ZnCl_2_ was synthesized by dissolving analytical grade cysteamine hydrochloride (0.02 mol, 2.27 g) with zinc sulfate heptahydrate (0.02 mol, 5.75 g) in double distilled water. The reaction pH of 6.5 was maintained by a 0.1 mol/L NaOH solution; the reaction was stirred and maintained at 90°C for 1 h. After filtration, a white powder was obtained, and the unreacted zinc sulfate heptahydrate was filtered. The products were dried at 80°C for 4 hours, and the ratio of the observed zinc(II) content with the stoichiometric amount of zinc(II) determined the yield at approximately 88.89%. Calcd. for (C_2_H_6_NS)_2_Zn·ZnCl_2_(%): Zn, 36.47; CS, 43.87. Observed (%): Zn, 35.73; cysteamine, 42.91. (C_2_H_6_NS)_2_Zn·ZnCl_2_ crystals were grown by dissolving a moderate amount of products (100 mg/mL) in a 50 mL beaker. The colorless filtrate was stored at room temperature for approximately one month, and the colorless flaky crystals of complex were obtained. The complex in polymer form was [ZnCl_2_{Zn(u-S-CH_2_CH_2_NH_2_)}].

### 2.3. X-Ray Diffraction Crystallography

The appropriate crystals were cut from larger crystals for X-ray diffraction on a Bruker Smart 1000-CCD diffractometer with graphite monochromated Mo K*α* radiation (*λ* = 0.71073 Å). The data were collected at 150 K. A colorless and transparent crystal with dimensions of 0.43 mm × 0.34 mm × 0.26 mm was fixed on a glass fiber. The structure was solved by direct methods (SHELXS-97) and refined by full-matrix least squares using SHELXS-97. All nonhydrogen atoms were obtained from the difference Fourier map, and full-matrix least-squares refinements on *F* ^2^ were carried out with anisotropic thermal parameters. Hydrogen atoms in the ligand were generated geometrically. The structure refinement parameters for the title complex are given in Table 1, and the crystallographic data are deposited with the Cambridge Crystallographic Data Centre under deposition number CCDC: 1010457.

## 3. Results and Discussion

### 3.1. X-Ray Crystal Structure Analysis

**Figure 1:**
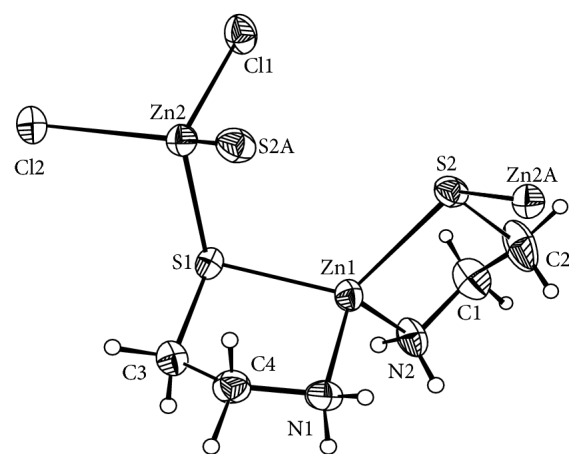
The molecular structure of compound 1 (ellipsoid parameter(s) 30%).

**Figure 2:**
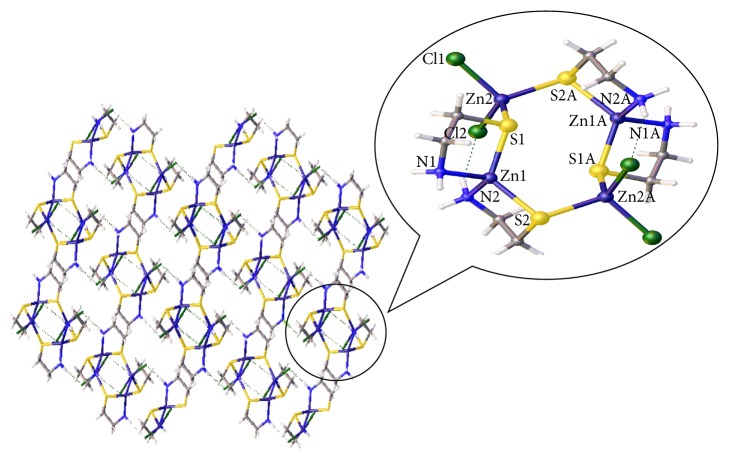
Crystal packing diagram of compound 1 (dotted lines indicate H bonding).

**Figure 3:**
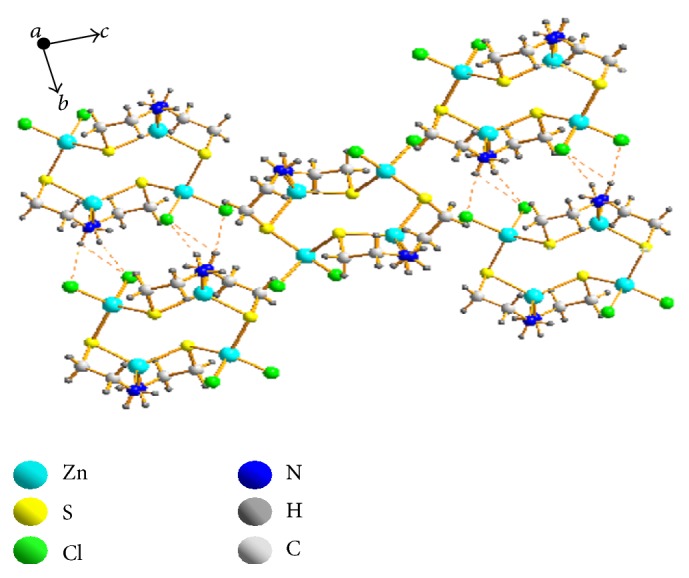
Crystal packing diagram of compound 1 (dotted lines indicate H bonding).

The single crystal X-ray diffraction analysis reveals that the complex [ZnCl_2_{Zn(u-S-CH_2_CH_2_NH_2_)}]*n* crystallises in a monoclinic system with space group *P* 2_1_/*n*. Crystallographic data and structure refinement parameters for the title complex are given in Table 1, and the selected bond distances and angles are shown in Table 2. The key fragments of the structures and the atom numbering are shown in Figure 1, and the crystal packing diagram of the complex is shown in Figures 2 and 3. The crystal consisted of an asymmetric unit that is an independent part of a unit cell consisting of two Zn^2+^ cations, two C_2_H_6_NS^−^ anions, and two chlorine anions. The coordination of Zn(1) was coordinated by two donor nitrogen atoms and bidentatelly by two sulphur atoms from two C_2_H_6_NS^−^ anions; the coordination polyhedron of Zn(2) was coordinated by two chlorine anions and two sulphur atoms. The Zn(II) ion of the plane formed a distorted trigonal bipyramidal configuration. The Zn(II) ion was four-coordinated by two amidogen nitrogen atoms (N1, N2) and two sulphur atoms (S1, S2) from the same ligand. The structure bridged a molecular zinc chloride (Zn2, Cl1, and Cl2) through the sulphur atom of cysteamine. The bond lengths of Zn1-S1, Zn1-N1, Zn1-S2, and Zn1-N2 ranged from 0.2024 to 0.2393 nm, which is a typical distance range for zinc(II) ion complex with C and N atoms. When comparing the bond lengths of Zn-N, Zn-S, and Zn-Cl, longer bond lengths had a stronger combining capacity between nonhydrogen atoms (N, S, and Cl) and zinc ions.

Tables 2 and 3 contain one type of hydrogen bond in the crystal of the zinc(II) complex: hydrogen bonds between the crystalline amidogen and chlorine atoms (N(2)-H(2D)⋯Cl(1)1, N(1)-H(1D)⋯Cl(2)2, N(1)-H(1D)⋯Cl(1)3, N(1)-H(1D)⋯Cl(2)2) which make the structure more stable.

### 3.2. FT-IR Spectra

**Figure 4:**
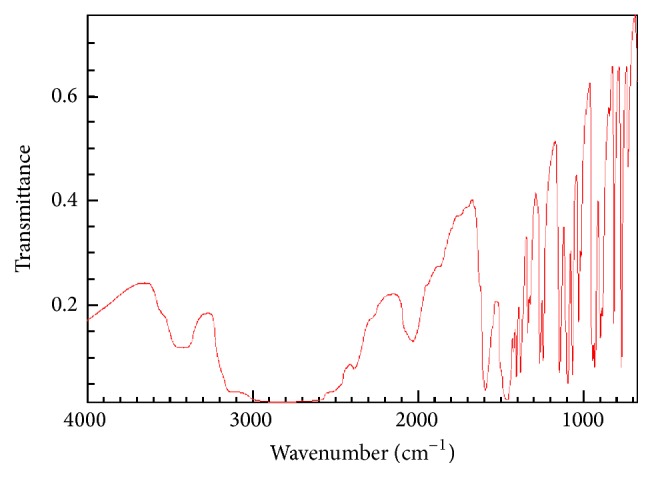
The standard FT-IR spectrum of 2-aminoethanethiol hydrochloride.

**Figure 5:**
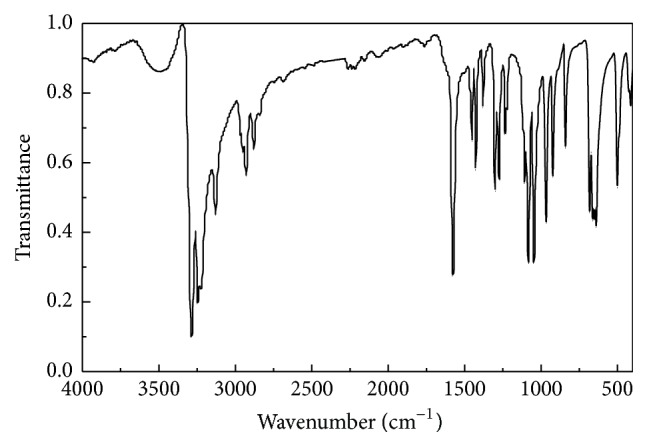
FT-IR spectrum of the complex.

The FT-IR spectra of the complex are shown in Figure 5. According to the infrared spectrum of cysteamine (Figure 4), after the reaction of 2-aminoethanethiol hydrochloride with zinc salt, the product in the main absorption peak clearly changed. Under acidic conditions, the end NH_2_ group of cysteamine easily formed -NH_3_^+^ and generated a wide absorption peak at 3000 cm^−1^; then the peaks observed from 2500 to 3000 cm^−1^ formed an ammonium band. Complex contains two narrow and medium absorption peaks at 3341 and 3269 cm^−1^ that were in good agreement with the N-H stretching vibration; this illustrates that NH_2_ in molecules participates in the coordination, and free -NH_3_^+^  does not exist. The S-H band of the ligand appears at 2507 cm^−1^, but the characteristic absorption peaks of the complex disappeared; the complex of cysteamine molecular ligand coordination may be consistent with zinc. According to the single crystal structure, FT-IR spectra were in good agreement with the experimental data.

### 3.3. Thermal Analysis

**Scheme 1:**
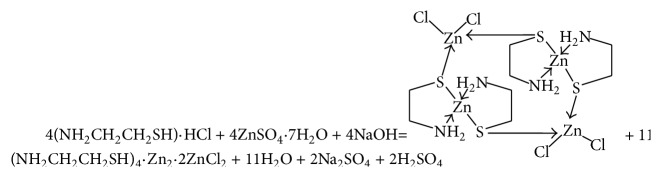
The process of the reaction.

**Figure 6:**
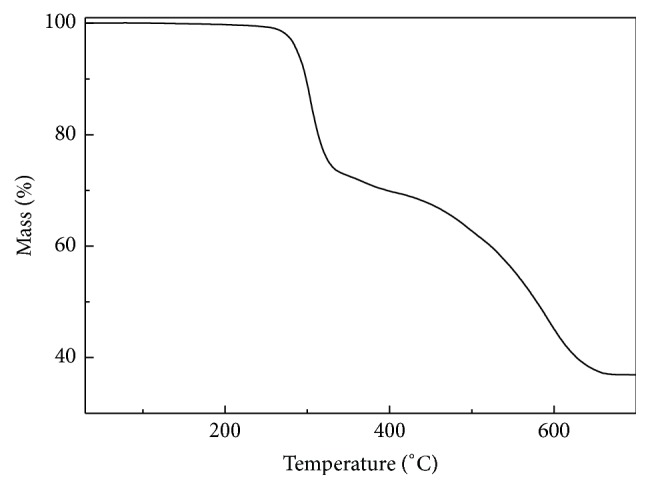
Thermogravimetric curve of the complex.

The thermal decomposition of complex can help elucidate the coordination structure of the complex. The TG curve of the complex is given in Figure 6, and the possible pyrolysis reaction and the experimental and calculated percent mass losses in the thermal decomposition process of the complex are summarized as follows (Scheme 1). The first mass loss of the compound occurred at approximately 290°C in the DG curve, suggesting that there was no crystalline water. This is consistent with the single crystal structure. Then loss at 290°C corresponds to oxidation and decomposition of the ligand. And it can be attributable to structure rearrangement or phase transformation in the solid complex. The thermal decomposition processes of the complex include oxidation and pyrolysis of the ligand, and the last residual quantity was 35.6% at temperature of 660°C.

### 3.4. UV-Vis

**Figure 7:**
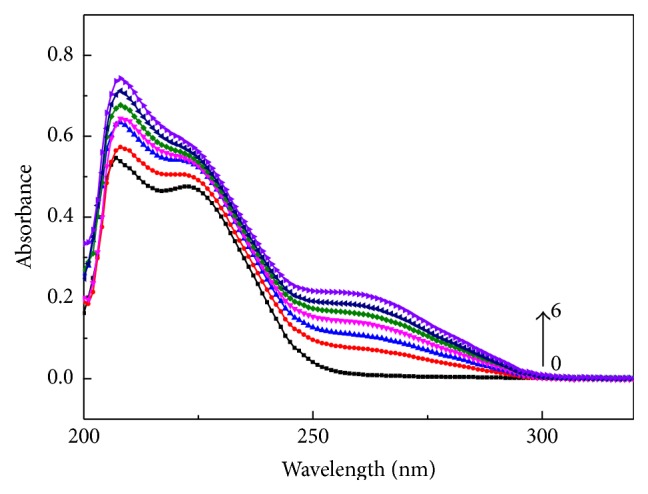
The UV-Vis absorption spectra of complex (1.7 × 10^−4^ mol/L) with Ct-DNA (from bottom to top, 0, 2 × 10^−5^ mol/L, 2.8 × 10^−5^ mol/L, 3.6 × 10^−5^ mol/L, 4.4 × 10^−5^ mol/L, 5.2 × 10^−5^ mol/L, and 6 × 10^−5^ mol/L).

Electronic absorption spectra are initially employed to study the blinding of complex to DNA. The interaction of Zn(II) complex with Ct-DNA has been monitored in Tris-HCl (pH 7.2) buffer solutions. The concentration of the complex is a stable value with the different concentration of Ct-DNA (0–6 × 10^−5^ mol/L). The UV studies reveal that the interaction causes “hyperchromism effect” of DNA, but no obvious bathochromic shift occurred (Figure 7), and hyperchromism results from the damage of DNA double-helix structure [18]. According to the experimental results, we speculated that the center zinc ions directly or indirectly combine with oxygen atom of phosphoric acid in the skeleton and offense dihydrogen phosphate ester bond, but base composition is not damaged. Zinc ions have the function of Lewis acid, when they are in coordination with the oxygen in dihydrogen phosphate ester bond, causing oxygen activation, easy to fracture [18]. The literatures reported the application of cysteamine, and we studied the interaction of zinc complex with Ct-DNA which can be tuned to control its reactivity in medical science and biological applications. The experimental results showed that the complex is bound to DNA intercalative (intercalation binding).

### 3.5. Viscosity Measurements

**Figure 8:**
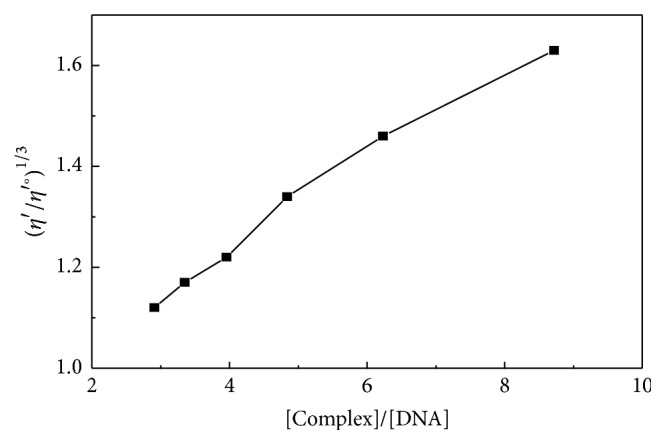
Effect of increasing amount of the complex on viscosities of Ct-DNA at 25°C ([Complex]/[DNA] = 2.91, 3.35, 3.96, 4.84, 6.23, and 8.72).

Spectroscopic data are necessary but not sufficient to support intercalation binding mode. Viscosity measurements being sensitive to length change of DNA are regarded as the important method for the classical intercalation model in solution. As a means for further clarifying the binding of this complex, we studied the viscosity on DNA by varying the concentration of the added complex. As it is observed from Figure 8 in the presence of the Zn(II) complex and Ct-DNA, the data of (*η*′/*η*′°)^1/3^ was increased with the increasing ratio of [Complex]/[DNA]. The separation of base pairs at intercalation sites and hence an increase in overall DNA length can be explained; the classical intercalation model demands that the DNA helix must lengthen as base pairs are separated to accommodate the bound ligand, leading to the increase of DNA viscosity [18].

## 4. Conclusions

In conclusion, new Zn(II) complex of (C_2_H_6_NS)_2_Zn·ZnCl_2_ was synthesized. The complex was investigated using several techniques, including elemental analysis, FT-IR, and TG. Thermogravimetric analysis evaluated the thermostability of the Zn(II) complex and this strategy offers a powerful tool for the preparation of Zn(II) complex with potential value in many industries. The UV hyperchromism of the absorption bands as well as the viscosity increase confirms that (C_2_H_6_NS)_2_Zn·ZnCl_2_ can strongly combine with Ct-DNA by intercalation binding.

## Figures and Tables

**Table 1:** Crystal data and structure refinement for the title complex.

Parameter	Character
Formula	C_8_H_24_Cl_4_N_4_Zn_4_S_4_
Formula weight	707.83
Crystal system, space group	Monoclinic, *P* 2_1_/*n*
*a*/nm	0.84294(4)
*b*/nm	0.83920(4)
*c*/nm	1.65787(8)
*α*/(°)	90.00
*β*/(°)	100.787(5)
*γ*/(°)	90.00
*D*/g/cm^3^	2.041
*F*(000)	704
Limiting indices	−9 ≤ *h* ≤ 10, −10 ≤ *k* ≤ 8, −18 ≤ *l* ≤ 20
Temperature/K	150
Wave/nm	0.071073
Volume/nm^3^	1.15205(10)
*Z*	2
GOF	1.121
*R* (*I* > 2*σ*(*I*))	*R*_1_ = 0.0266, *wR*_2_ = 0.0610
*R* (all data)	*R*_1_ = 0.0325, *wR*_2_ = 0.0636
Largest diff. peak and hole e/nm^3^	688 and −619

**Table 2:** Bond lengths (nm) and bond angles (°) of the title complex.

Bond	Dist./nm
Zn(1)-N(1)	0.2024(3)
Zn(1)-N(2)	0.2039(3)
Zn(2)-Cl(1)	0.22831(9)
Zn(2)-Cl(2)	0.22669(9)
C(1)-N(2)	0.1480(4)
C(2)-C(1)	0.1515(4)
Zn(1)-S(1)	0.23093(8)
Zn(1)-S(2)	0.23069(8)
Zn(2)-S(1)	0.23405(8)
Zn(2)-S(2)	0.23522(8)
C(2)-S(2)	0.1842(3)

Angle	(°)

N(1)-Zn(1)-N(2)	110.44(11)
N(1)-Zn(1)-S(2)	122.17(8)
N(2)-Zn(1)-S(2)	90.48(8)
N(2)-Zn(1)-S(1)	121.61(8)
N(1)-Zn(1)-S(1)	91.90(8)
S(1)-Zn(1)-S(2)	122.45(3)
Cl(1)-Zn(2)-Cl(2)	112.04(3)
Cl(2)-Zn(2)-S(1)	107.52(3)
Cl(1)-Zn(2)-S(1)	105.14(3)
Cl(2)-Zn(2)-S(2)	104.76(3)
Cl(1)-Zn(2)-S(2)	113.52(3)
S(1)-Zn(2)-S(2)	113.86(3)

**Table 3:** Hydrogen bond lengths (nm) and bond angles (°) of the title complex.

D-H⋯A	*d*(D-H)/nm	*d*(H⋯A)/nm	*d*(D⋯A)/nm	D-H⋯A (°)
N(2)-H(2D)⋯Cl(1)^1^	0.090	0.265	0.3465(3)	150.6
N(1)-H(1D)⋯Cl(2)^2^	0.090	0.272	0.3339(3)	127.0
N(1)-H(1D)⋯Cl(1)^3^	0.090	0.291	0.3367(3)	113.4
N(1)-H(1E)⋯Cl(1)^4^	0.090	0.250	0.3343(3)	157.0

Symmetry codes: 1 = *x*, *y* + 1, *z*; 2 = −*x* + 1/2, *y* + 1/2, −*z* + 3/2; 3 = *x*, *y* + 1, *z*; 4 = *x*, *y* + 1, *z*.
